# Complete Genome Sequence of Highly Virulent Aeromonas hydrophila Strain D4, Isolated from a Diseased Blunt-Snout Bream in China

**DOI:** 10.1128/MRA.01035-18

**Published:** 2019-01-24

**Authors:** Lei Zhu, Jin-Shui Zheng, Wei-Min Wang, Yi Luo

**Affiliations:** a College of Fisheries, Key Laboratory of Freshwater Animal Breeding, Ministry of Agriculture, Huazhong Agricultural University, Wuhan, Hubei, People’s Republic of China; b State Key Laboratory of Agricultural Microbiology, College of Life Science and Technology, Huazhong Agricultural University, Wuhan, Hubei, People’s Republic of China; c Freshwater Aquaculture Collaborative Innovation Center of Hubei Province, Wuhan, Hubei, People’s Republic of China; University of Delaware

## Abstract

Aeromonas hydrophila is the causative agent of motile aeromonad septicemia. Here, we present the complete genome sequence of highly virulent A. hydrophila strain D4, which was isolated from a diseased blunt-snout bream in China. It comprises one circular chromosome and four previously unreported plasmids with a total length of 5,275,132 bp.

## Abstract (precis)

Aeromonas hydrophila is the causative agent of motile aeromonad septicemia. Here, we present the complete genome sequence of highly virulent A. hydrophila strain D4, which was isolated from a diseased blunt-snout bream in China.

## ANNOUNCEMENT

The Gram-negative bacterium Aeromonas hydrophila is ubiquitous in various aquatic environments (1). It is the etiological agent of motile aeromonad septicemia (MAS) in fish, a severe disease that has frequently caused huge economic losses in the cyprinid fish industry worldwide (2). Since 2009, MAS outbreaks have occurred in western Alabama (USA), leading to the loss of more than three million pounds (ca. 1,360 metric tons) of channel catfish each year (3). In China, MAS has been a prominent problem in aquaculture over the past 30 years (4). Even though some virulence factors have been identified (5), the multifactorial mechanisms underlying A. hydrophila pathogenesis and immune evasion are poorly understood (6). A. hydrophila strain D4 was isolated from a diseased blunt-snout bream (Megalobrama amblycephala) at a fish farm in Wuhan City, Hubei Province, China, in 2012. The tissue homogenate of the liver of the diseased blunt-snout bream was made with 0.85% (wt/vol) saline (sodium chloride; tissue to saline ratio, 1:5). After centrifugation at 3,000 rpm for 10 min, the supernatant was aseptically inoculated on LB agar and incubated at 28°C for 24 h. Many single colonies were obtained. Strain D4 was a randomly picked colony that was initially identified as A. hydrophila via biochemical profiling and 16S rRNA gene sequencing, and its pathogenicity was validated by a recursive infectivity experiment in blunt-snout bream (7). Here, to reveal the virulence differences at the genomic level for developing effective control strategies, the complete genome sequence of A. hydrophila strain D4 was determined in this study. For bacterial genomic DNA extraction, bacterial cells were incubated in LB culture medium at 28°C until the optical density at 600 nm (OD_600_) reached 0.6, and the DNA was extracted using an EasyPure genomic DNA kit (Beijing TransGen Biotech Co., Ltd.). About 3 μg DNA were used for the library preparation with a Nextera XT DNA library prep kit (Illumina, USA).

The genome sequencing of A. hydrophila strain D4 was performed on an Illumina HiSeq 2500 instrument, using a 500-bp paired-end genomic library and 150-nucleotide length reads. *De novo* assembly was performed using the ABySS alignment tool version 1.5.4 (8), with *k*-mers of 21 to 41. Raw sequencing data were quality trimmed using Trimmomatic v0.32 (ILLUMINACLIP, TruSeq3-PE.fa:2:30:10; LEADING, 3; TRAILING, 3; SLIDINGWINDOW, 4:15; MINLEN, 30; AVGQUAL, 20) (9). Based on 7,705,332 pairs of the filtered reads, a total of 36 contigs (>500 bp) were obtained by preliminary assembly, and the coverage was 219-fold. The gaps in the scaffolds were closed by GapCloser v1.12 (maximum read length, 100; overlap parameter, 25) (http://sourceforge.net/projects/soapdenovo2/files/GapCloser). Different scaffolds were connected by reverse PCR and subsequent Sanger sequencing. Genome annotation was performed using the NCBI Prokaryotic Genome Automatic Annotation Pipeline (PGAAP) (10) with default parameters.

The complete genome of A. hydrophila strain D4 comprises one circular chromosome of 5,100,520 bp and four circular plasmids, namely, pAhD4-1 (156,086 bp), pAhD4-2 (6,318 bp), pAhD4-3 (6,163 bp), and pAhD4-4 (6,045 bp). It contains 4,774 genes, including 174 genes in plasmids. A total of 117 tRNAs and 31 rRNA operons were predicted by PGAAP (see Table 1). The large plasmid pAhD4-1 showed partial homology with plasmid 4 of Aeromonas salmonicida subsp. salmonicida A449 (166,749 bp, NCBI accession number CP000645). Plasmid pAhD4-1 was found to carry a high-persistence (Hip) toxin-antitoxin system (toxins, *hipA* and *hipB*; antitoxins, AhyD4_23105 and AhyD4_23110), which could underlie antibiotic tolerance and recalcitrant chronic bacterial infections (11).

**TABLE 1:** Genome features of Aeromonas hydrophila D4

Feature	Result for chromosome	Result for plasmid:			
		pAhD4-1	pAhD4-2	pAhD4-3	pAhD4-4
Length (bp)	5,100,520	156,086	6,318	6,163	6,045
G+C content (%)	60.8	50.1	56.3	54.3	51.5
No. of CDS^*a*^	4,600	152	8	7	7
No. of tRNAs	117				
No. of rRNAs	31				
Accession no.	CP013965	CP013966	CP013967	CP013968	CP013969

a CDS, coding sequences.

The availability of the D4 genome contributes to the understanding of A. hydrophila pathogenesis during infection, environmental adaptations, and horizontal gene transfer among the Aeromonas spp.

### Data availability.

This complete genome has been deposited at DDBJ/EMBL/GenBank under the accession numbers CP013965 to CP013969 (BioProject accession number PRJNA308632). Sequence data have been submitted to SRA under the accession number SRP165140.
